# Patterns of microRNA Expression in Non-Human Primate Cells Correlate with Neoplastic Development *In Vitro*

**DOI:** 10.1371/journal.pone.0014416

**Published:** 2010-12-22

**Authors:** Belete Teferedegne, Haruhiko Murata, Mariam Quiñones, Keith Peden, Andrew M. Lewis

**Affiliations:** 1 Laboratory of DNA Viruses, Division of Viral Products, Center for Biologics Evaluation and Research, Food and Drug Administration, Bethesda, Maryland, United States of America; 2 Laboratory of Retrovirus Research, Division of Viral Products, Center for Biologics Evaluation and Research, Food and Drug Administration, Bethesda, Maryland, United States of America; 3 Bioinformatics and Computational Biosciences Branch, National Institutes of Health, Bethesda, Maryland, United States of America; University of Texas M. D. Anderson Cancer Center, United States of America

## Abstract

MicroRNAs (miRNAs) are small noncoding RNAs that negatively regulate gene expression post-transcriptionally. They play a critical role in developmental and physiological processes and have been implicated in the pathogenesis of several diseases including cancer. To identify miRNA signatures associated with different stages of neoplastic development, we examined the expression profile of 776 primate miRNAs in VERO cells (a neoplastically transformed cell line being used for the manufacture of viral vaccines), progenitor primary African green monkey kidney (pAGMK) cells, and VERO cell derivatives: spontaneously immortalized, non-tumorigenic, low-passage VERO cells (10-87 LP); tumorigenic, high-passage VERO cells (10-87 HP); and a cell line (10-87 T) derived from a 10-87 HP cell tumor xenograft in athymic nude mice. When compared with pAGMK cells, the majority of miRNAs were expressed at lower levels in 10-87 LP, 10-87 HP, and 10-87 T cells. We identified 10 up-regulated miRNAs whose level of expression correlated with VERO cell evolution from a non-tumorigenic phenotype to a tumorigenic phenotype. The overexpression of miR-376a and the polycistronic cluster of miR-376a, miR-376b and miR-376c conferred phenotypic changes to the non-tumorigenic 10-87 LP cells that mimic the tumorigenic 10-87 HP cells. Thirty percent of miRNAs that were components of the identified miRNAs in our spontaneously transformed AGMK cell model are also dysregulated in a variety of human tumors. These results may prove to be relevant to the biology of neoplastic development. In addition, one or more of these miRNAs could be biomarkers for the expression of a tumorigenic phenotype.

## Introduction

Neoplastic development represents cumulative genetic and epigenetic events leading to the emergence of cells that can attain a tumorigenic phenotype [1], [2], [3], [4]. Neoplastic transformation of cells cultured *in vitro* can be induced by several methods, such as treatment with chemical carcinogens or radiation, viral infection, or the introduction of oncogenes [1], [2], [5]. To help understand how tumors originate and progress in mammals, cells transformed *in vitro* by these methods have been used for many years to study processes analogous to neoplastic development *in vivo*.

Cells from non-human mammalian species, in the absence of known carcinogenic stimuli, can spontaneously undergo processes of neoplastic transformation during culture *in vitro*. It is well documented that repeated passage of cells in culture can result in their immortalization and can lead to the acquisition of the capacity to form tumors when injected into animals [6], [7]. The neoplastically transformed VERO cell line, which was derived from primary African green monkey kidney (pAGMK) cells [8] and is used in diagnostic virology and for the manufacture of viral vaccines, is known for its propensity to develop the capacity to form tumors following prolonged *in vitro* passage [9], [10], [11], [12]. In our studies, we have shown that the 10-87 VERO cell line was non-tumorigenic at low passage [passage (p) 148] when injected into athymic nude mice. However, when these cells were serially passaged in culture to higher passage levels (p256), they were found to be tumorigenic when injected into newborn nude mice [11]. Thus, the neoplastic processes that occur spontaneously in VERO cells in culture, resulting in cells that express a tumorigenic phenotype, offer an opportunity to evaluate the molecular differences that may underlie the different stages of neoplastic development *in vitro*.

Recently, it has become evident that, in addition to mutation of, and/or changes in, the expression of protein-coding genes, alterations in the expression of non-coding microRNAs (miRNAs) can also contribute to cancer pathogenesis [13], [14], [15], [16]. miRNAs modulate gene expression at the post-transcriptional level by inducing either degradation of the mRNA transcript or translational repression [17]. Many of the genes for these targeted mRNAs are involved in proliferation, differentiation, and apoptosis [13], [14], [15], [17], [18], [19], processes that are frequently altered during neoplastic development. Indeed, it has been shown that dysregulation of miRNA expression can contribute to cellular transformation and tumorigenesis [13], [14], [20]. To identify the miRNAs involved in these processes, a number of studies have compared miRNA expression profiles of various tumors with those of corresponding normal tissues [20], [21], [22]. These studies revealed that alterations in miRNA expression profiles can serve as molecular signatures of particular types of cancer. Importantly, miRNA expression profiles appear to be more informative than mRNA expression profiles in reflecting the developmental lineage and the differentiation state of tumors [20], [22]. Furthermore, profiling of miRNA expression can identify miRNAs that might serve as diagnostic and prognostic markers for different cancers as well as potential therapeutic targets [23], [24], [25], [26], [27]. Based on the pleiotropic effects of miRNAs in neoplastic development, we hypothesized that the molecular events that occurred during serial passage of pAGMK cells in tissue culture that led to immortalization and then to the expression of a tumorigenic phenotype might be associated with progressive alterations of cellular miRNA regulatory networks analogous to those observed in neoplastic cells arising *in vivo*.

In this report, we performed high-throughput miRNA profiling to examine the expression level of miRNAs in pAGMK cells and in VERO cells at non-tumorigenic and tumorigenic stages of neoplastic development. The analysis involved (1) pAGMK cells, (2) non-tumorigenic 10-87 low-passage VERO cells (10-87 LP), (3) tumorigenic, high-passage VERO cells (10-87 HP), and (4) a cell line (10-87 T) derived from a 10-87 HP cell tumor xenograft in athymic nude mice. Our data revealed that, although some miRNA dysregulation occurred during the immortalization of the AGMK cells that established the VERO cell line, the majority of miRNA dysregulation occurred in VERO cells during the transition from a non-tumorigenic to a tumorigenic phenotype. We identified the up-regulation of specific miRNAs that correlated with, and were perhaps involved in, the evolution of the neoplastic phenotype that occurred during serial passage of VERO cells in culture. Indeed, the overexpression of miR-376a and the polycistronic cluster of miR-376a, miR-376b and miR-376c in non-invasive 10-87 LP cells were able to recapitulate the migration and invasion phenotypes of 10-87 HP cells. Cumulatively, these data suggest that the patterns of dysregulation of the miRNAs identified in this study might be a feature of neoplastic development *in vitro* in kidney cells from this non-human primate.

## Results

### *In vitro* cell migration and invasion activities of VERO cell lines

The 10-87 VERO cell lines used in this study were derived from the World Health Organization (WHO) VERO cell bank (10-87) after serial passage in tissue culture from p140 to p256 [11]. The non-tumorigenic 10-87 LP cells (p148), the tumorigenic 10-87 HP cells (p256), and the VERO tumor cell line 10-87 T, which was derived from a tumor xenograft formed by the inoculation of 10-87 HP cells into newborn athymic nude mice [11], were selected for study. The characteristics of the cell lines used in this study are summarized in Table 1.

**Table 1:** Growth rates and tumorigenic characteristics of the cell lines used for miRNA studies.

Cell Line(passage level)	Doubling Time (h) 1	Tumor Incidence in Nude Mice		Reference
		Newborns	Adults	
10-87 LP (p148)	25	0/17^2^	0/14^2^	[11]
10-87 HP (p256)	26	16/30^2^	0/18^2^	[11]
10-87 T	25	ND	ND	[11]
A4497 (p165)	18	0/4^2^	2/25^2^	[11]
A4497 (p>200)	ND	12/13	6/10	[11]
SF- VERO	20	9/17^2^	4/17^2^	[11]
CV-1 (p40)	27	0/7^3^	0/10^3^	
BSC-1 (p49)	30	0/15^3^	0/10^3^	

1 Cells (5×10^4^) were seeded in 60-mm dishes, and viable cell numbers were determined in triplicate by trypan-blue exclusion after 16, 24, 48, and 72 h. Cell-doubling times were estimated from growth-curve plots.

2 Observation period was 1 year in adult and newborn nude mice.

3 Newborns have been observed for >8 months and adults have been observed for 1 year.

ND: not determined.

**Figure 1:**
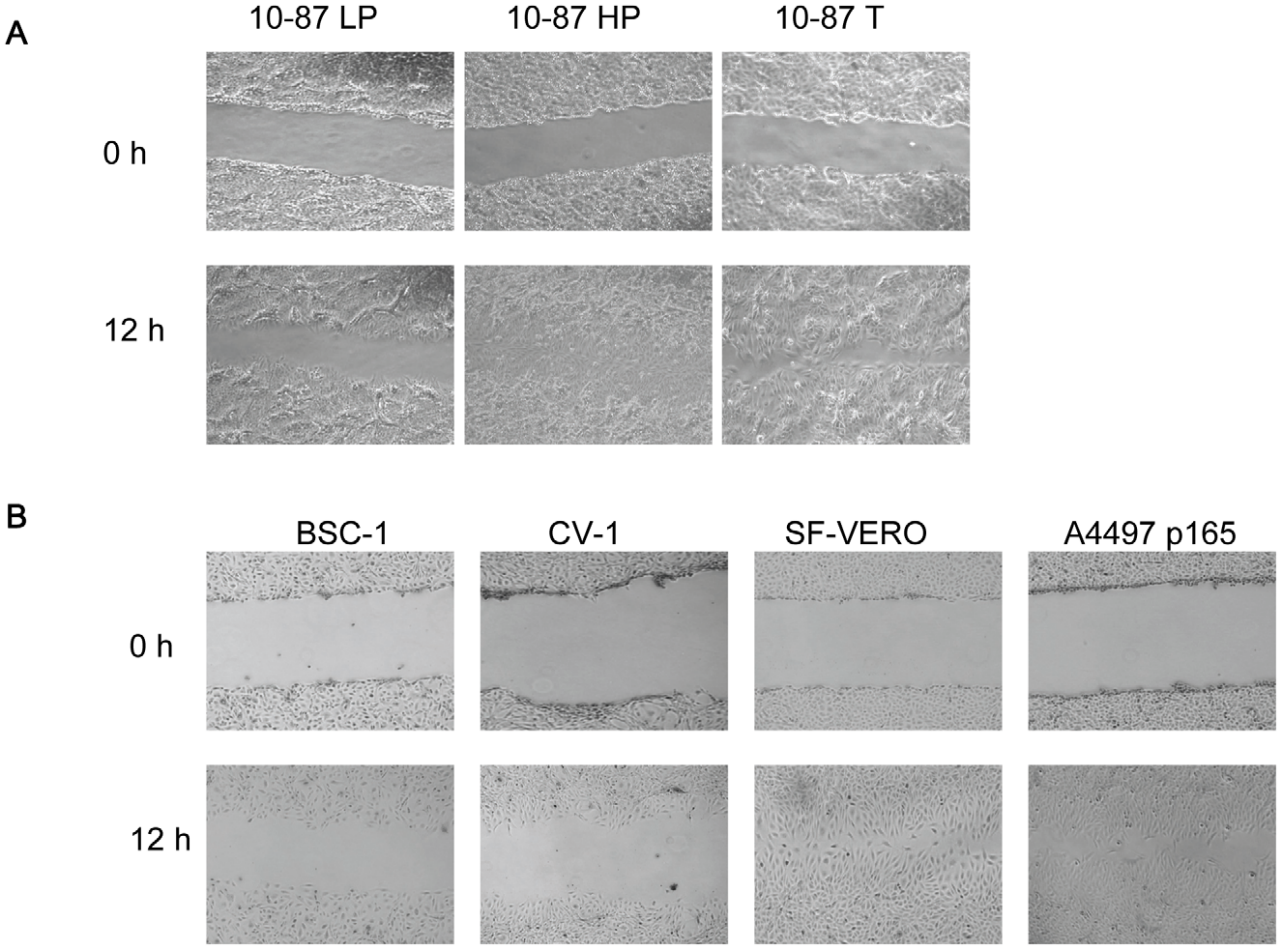
Wound-healing assay using African green monkey kidney cell lines. (A) 10-87 LP, 10-87 HP and 10-87 T. (B) BSC-1, CV-1, SF-VERO, and A4497 (p165). Microscopic observations were recorded at 0, 6, 9, and 12 h after scratching the cell surface; the 0 and 12 hour images are shown.

**Figure 2:**
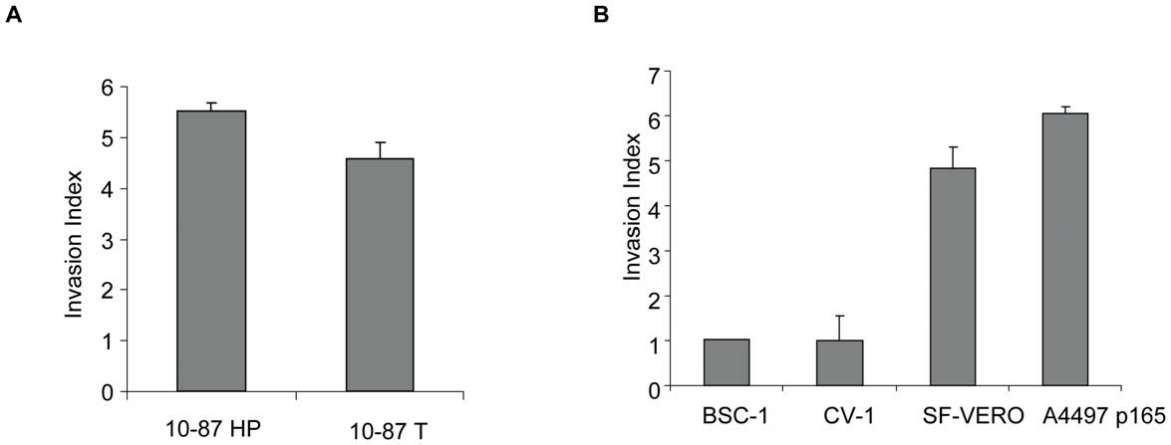
Matrigel-invasion assay with African green monkey kidney lines. (A)10-87 HP and 10-87 T; (B) BSC-1, CV-1, SF-VERO, and A4497. The cells were placed in a Matrigel-invasion chamber, and cells that invaded the Matrigel were fixed, stained, and counted. Test and control (10-87 LP) cells that invaded the Matrigel were counted in 5 fields under a 20× objective. The data were expressed as fold percent invasion of 10-87 LP cells. The mean data are plotted with their standard deviations.

Survival, proliferation, invasion, and migration are among the common functions acquired by cancer cells during neoplastic development [28], [29], [30], [31]. To initiate the characterization of our AGMK cell lines, and to evaluate the impact of serial passage on proliferation, invasion, and migration, we compared the different VERO cell lines by cell-growth rates, wound-healing migration assays, and invasion assays. The growth rates (cell doubling times) of the 10-87 cells were comparable, indicating that serial passage did not affect the rate of cell proliferation in these cells (Table 1). The wound-healing assay measures cell migration/motility *in vitro*. Figure 1A shows representative photomicrographs taken of wounded cell-culture monolayers at 0 and 12 h after the monolayer surfaces were scratched. The 10-87 HP cells and 10-87 T cells displayed an increase in migration compared with 10-87 LP cells. Little or no motility was observed in 10-87 LP cells during this observation period, whereas the 10-87 HP cells and 10-87 T cells started to fill in the wound as early as 9 h (data not shown). In the invasion assay, the Matrigel matrix serves as a barrier to distinguish between invasive cells and non-invasive cells. In this assay, the relative migration of cells from the upper chamber to the lower chamber compared with a reference cell line (non-tumorigenic 10-87 LP) provides an invasion index. We found that 10-87 HP cells and 10-87 T cells had a more than four-fold increase in invasiveness over 10-87 LP cells (Fig. 2A).

The above results suggested: first, 10-87 LP cells had already attained the proliferative potential expressed by 10-87 HP and 10-87 T cells (Table 1); second, serial passage of VERO cells in tissue culture beyond the 10-87 LP p140 level resulted in the acquisition of the potential to migrate and invade; third, migration and invasion phenotypes of 10-87 HP and 10-87 T cells appeared to correlate with their ability to form tumors *in vivo*.

### miRNA expression patterns in VERO cells expressing different phenotypes

**Figure 3:**
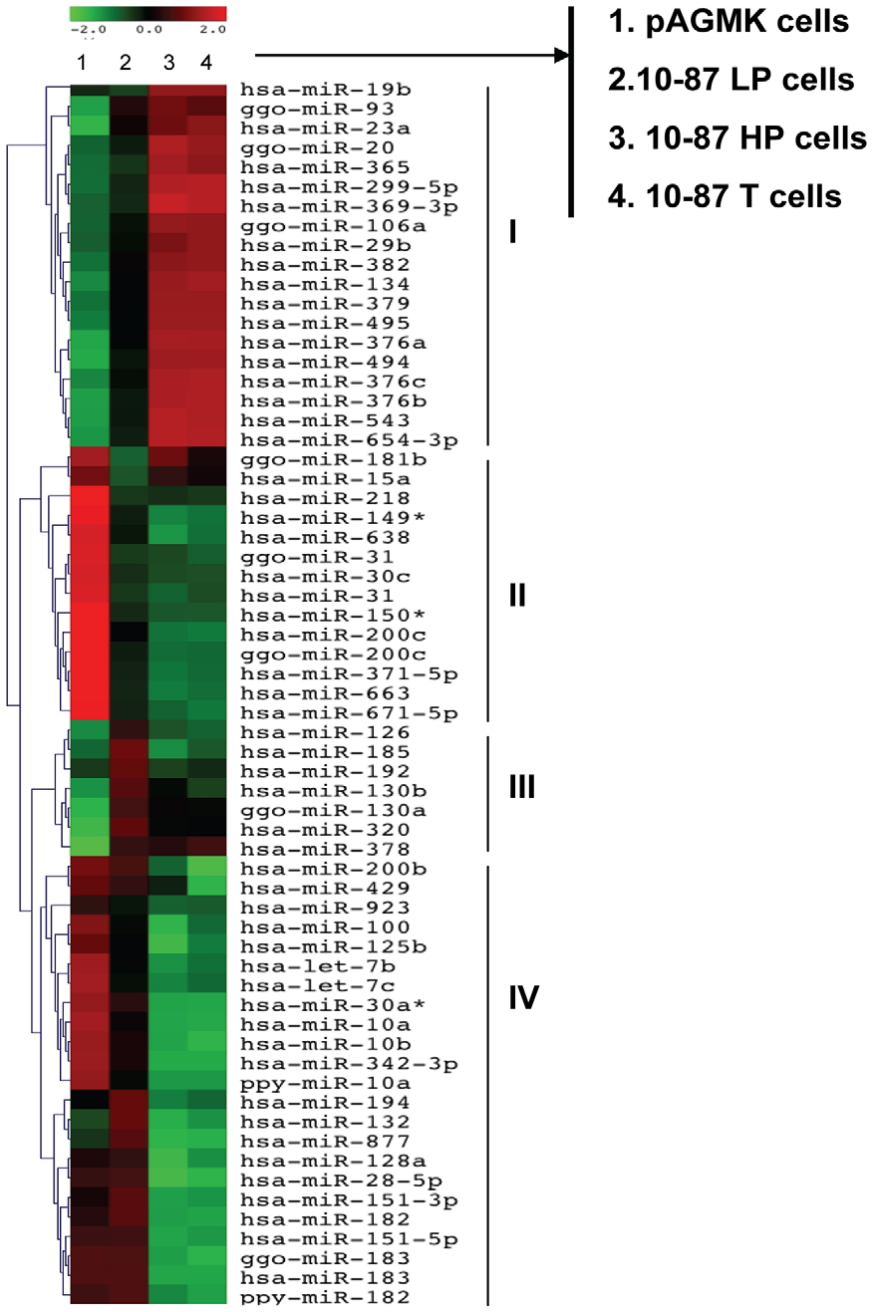
Hierarchical clustering of miRNA expression. miRNAs included in the heat-map had a fold change >±2 and were significantly expressed (*p*<0.01). Each row shows the relative expression level for a single miRNA and each column represents miRNA profiles of the average triplicate array data: (1) pAGMK cells; (2) 10-87 LP cells; (3) 10-87 HP cells; (4) 10-87 T cells. The red or green color indicates relative high or low expression, respectively. Expression clusters representing different patterns of up-regulation to down-regulation are depicted by Roman numerals on the right hand side of the Figure.

The dysregulation of miRNA expression has been observed in cells obtained from various cancers [13], [20], [22]. Because the serial passage of VERO cells results in the acquisition of a tumorigenic phenotype [11], we evaluated whether miRNA expression could be correlated with the evolution of the neoplastic processes that were occurring in VERO cells during serial tissue-culture passage. Array-based miRNA profiling was performed on pAGMK cells and on non-tumorigenic and tumorigenic VERO cells. For each cell type, the analysis was performed in triplicate. Out of 776 primate miRNAs assayed, 654 were detected above background levels in at least 3 of the 12 datasets. When unsupervised hierarchical clustering analysis was done on the 654 miRNAs expressed in pAGMK cells, 10-87 LP cells, 10-87 HP cells, or 10-87 T cells, the miRNA tree generated was able to discriminate among all four types of cells, with pAGMK cells located at a distance from the clusters of the three VERO cell line derivatives (data not shown). ANOVA was done using the four cell types. All significantly expressed miRNAs with mean intensity values of more than 500 (arbitrary units) in at least in one of the four cell types and a *p*<0.01 were selected and transformed to z-intensity values for inclusion in the heat-maps. These miRNAs were regarded as significant and were considered to be differentially expressed (Fig. 3). Four distinct clusters of miRNAs were evident according to their similar expression patterns. The expression profile of miRNA in cluster-I indicated a general up-regulation in tumorigenic 10-87 HP cells and 10-87 T cells compared with pAGMK cells and 10-87 LP cells. Clusters-II,-III, and -IV contained miRNAs that were down-regulated in 10-87 HP cells and 10-87 T cells. Expression readouts of the miRNA arrays were subjected to paired, volcano-plot analysis to arrive at differences of means for each miRNA in 10-87 LP cells compared with 10-87 HP cells and 10-87 T cells, and 10-87 HP cells compared with 10-87 T cells. The analysis revealed distinct patterns of miRNA expression that distinguished the non-tumorigenic 10-87 LP cells from the tumorigenic 10-87 HP cells and 10-87 T cells (data not shown). When this comparison was performed between the miRNA expression profiles of 10-87 HP cells and 10-87 T cells, no significant differences were apparent (Table 2). These observations indicated that the dysregulation of miRNA expression during *in vitro* passaging correlated with the conversion of 10-87 LP cells to a tumorigenic phenotype at higher passage levels. Furthermore, no additional dysregulation of miRNA expression appeared to occur during tumor formation.

**Table 2:** Differentially expressed mature miRNAs in low-passage and high-passage VERO cells in comparison with pAGMK cells.

Name a	10-87 LP		10-87 HP		10-87 T		Chromosomal location	
	FC	*p*-value (*t*-test)	FC	*p*-value (*t*-test)	FC	*p*-value (*t*-test)	Human	Rhesus
miR-376a	-	-	8.1	2.35E-03	8.1	2.13E-03	14	7
miR-654-3p	-	-	7.1	2.08E-03	6.9	1.97E-03	14	7
miR-376c	-	-	6.6	2.08E-03	6.6	1.87E-03	14	7
miR-369-3p	-	-	6.3	4.65E-04	6.1	1.27E-03	14	7
miR-543	-	-	5.8	6.50E-04	5.6	1.14E-03	14	7
miR-376b	-	-	5.8	6.39E-03	8.4	1.19E-03	14	7
miR-494	-	-	4.7	4.67E-03	4.7	4.73E-03	14	7
miR-299-5p	-	-	4.5	3.68E-03	4.6	4.17E-03	14	7
miR-382	-	-	3.6	6.59E-03	3.7	6.29E-03	14	7
miR-134	-	-	3.2	3.82E-03	3.4	2.83E-03	14	7
miR-923	-	-	-2.2	4.47E-03	−2.1	1.03E-03	17	?
miR-374b	-	-	−2.2	7.04E-03	−2.2	4.19E-03	X	X
miR-342-3p	-	-	−2.2	2.98E-03	−2.2	4.39E-03	14	7
miR-200b	-	-	−2.3	8.53E-04	−3.3	2.39E-02	1	?
miR-671-5p	-2.1	2.96E-03	−2.7	6.32E-03	−2.9	5.79E-03	7	3
miR-10b	-	-	−4.0	6.93E-03	−4.3	4.32E-03	2	12
miR-125b	-	-	−3.7	4.86E-03	−2.9	5.08E-03	11	14
miR-100	-	-	−3.8	4.13E-03	−2.8	7.58E-03	11	14
miR-10a	−2.7	4.54E-03	−5.6	5.23E-03	−5.5	1.55E-03	17	16
miR-638	−3.4	1.24E-04	−5.4	2.58E-04	−4.7	1.16E-03	19	19
miR-150	−4.9	1.55E-05	−5.8	1.76E-03	−5.8	4.98E-04	19	19
miR-149	−4.3	6.52E-07	−6.0	5.83E-03	−5.7	6.07E-03	2	12
miR-371-5p	−5.1	1.64E-03	−6.8	8.13E-04	−6.5	1.04E-03	19	19
miR-663	−5.2	3.58E-07	−6.9	5.74E-05	−6.6	2.59E-03	20	20
miR-218	−6.9	9.04E-04	−7.0	1.17E-03	−7.2	9.22E-04	4	1
miR-200c	−5.4	7.13E-08	−8.0	1.33E-04	−8.1	1.18E-03	12	11
miR-31	−8.4	5.51E-11	−9.6	2.07E-04	−9.4	1.19E-04	9	15

FC: Fold change (log_2_) over pAGMK cells.

a only miRNAs that meet the criteria of being at least 4-fold up- or down-regulated with *p*<0.01.

### Identification of differentially expressed miRNAs

The next step was to identify miRNAs that were differentially expressed between pAGMK and either non-tumorigenic or tumorigenic VERO cells. Since it is likely that pAGMK cells are representative of the normal diploid precursor cell type that gave rise to VERO cells, they can serve as a baseline to identify the alterations in miRNA expression that occurred during serial passage of VERO cells in tissue culture. When comparing two groups, findings were considered significant if: (1) fold change was ≥4 relative to levels observed in pAGMK cells; (2) *t*-test *p-*value was <0.01, and (3) differences in fluorescence intensity between the means of two groups were >500 arbitrary units. The stringency was set at ≥4 fold with the intent of identifying the smallest number of miRNAs with the highest probability of contributing to VERO cell neoplastic development. Using these criteria, we identified 10 upregulated and 17 downregulated miRNAs in 10-87 HP cells (Table 2). The differentially expressed miRNAs in 10-87 T cells were the same as those in 10-87 HP cells. The 10 miRNAs that were down-regulated in 10-87 LP cells relative to pAGMK cells were a sub-set of the miRNAs whose expression was also down-regulated in the 10-87 HP cells and 10-87 T cells (Table 2). The magnitude of change in expression levels for these miRNAs was greater in 10-87 HP cells and 10-87 T cells when compared with 10-87 LP cells, demonstrating a progressive alteration of miRNA expression during serial passage of 10-87 LP cells and suggesting that their changed expression may be related to the acquisition of the new phenotype.

### Validation of miRNA expression

In order to validate the miRNA microarray data for VERO cells at the passage levels tested, we performed qRT-PCR analysis to quantify the expression level of selected miRNAs from a list of differentially expressed miRNAs. The data are presented as fold over pAGMK miRNA levels (Table 3). qRT-PCR analysis confirmed that miR-376a, miR-654-3p, miR-543, miR-382, miR-31, miR-200c, miR-218, and miR-183 paralleled the microarray miRNA levels. Included as controls were two miRNAs, miR-103 and miR-25, whose microarray expression levels were not statistically different in cell lines with different phenotypes. These miRNAs also showed no significant differences by qRT-PCR in 10-87 LP cells, 10-87 HP cells, and 10-87 T cells. These qRT-PCR results demonstrated that the microarray analysis accurately reflected expression levels of the miRNAs.

**Table 3:** Validation of the differential expression of selected miRNA in 10-87 VERO cells using Taqman quantitative RT-PCR^1^.

miRNA	10-87 LP	10-87 HP	10-87 T
miR-376a	2.2	27.7	24.6
miR-654-3p	1.8	17.8	8.9
miR-543	2.5	23.1	22.0
miR-382	1.2	10.3	10.9
miR-31	−435.0	−500.0	−625.0
miR-200c	−18.0	−18.7	−25.2
miR-218	−33.0	−20.0	−25.0
miR-183	−5.0	−9.1	−8.3
miR-103	1.0	1.1	1.0
miR-25	1.0	1.0	1.0

1 Results from the triplicate samples were averaged following normalization to the levels of small RNA U6B. The data were presented as fold change over pAGMK miRNA levels.

### miRNA expression patterns correlated with the level of neoplastic progression in other AGMK cell lines

The results above raised the question of whether the observed miRNA expression signature in 10-87 HP and 10-87 T cells was specific to this VERO lineage or was a more general phenomenon that might be associated with the acquisition of tumorigenic phenotypes by other cell lineages derived from the VERO cell line as well as from other immortalized AGMK cell lines. To address this question, we extended the qRT-PCR expression analysis of selected miRNAs to SF-VERO cells, a tumorigenic line of VERO cells adapted to grow in serum-free medium, and to A4497 VERO cells, a tumorigenic line of VERO cells developed independently of 10-87 VERO cells at the National Institutes of Health (Table 1). When the ability of the SF-VERO and A4497 VERO cell lines to migrate and invade in the *in vitro* assays was compared, a close similarity with the behavior of 10-87 HP cells was observed in the migration assay (Fig. 1B) and in the invasion assay (Fig. 2B). To determine if the similar phenotypes correlated with similar alterations in miRNA expression in these cell lines, a qRT-PCR analysis was done on the selected miRNAs (based on ≥4-fold changes at *p*<0.01). As found for 10-87 HP cells and 10-87 T cells, SF-VERO cells and A4497 VERO cells expressed increased levels of miR-376a, miR-654-3p, miR-543, and miR-382 over the levels found in pAGMK cells (Table 4). Similarly, the miRNAs down-regulated in 10-87 HP cells and 10-87 T cells, such as miR-31, miR-200c, miR-218, and miR-183, were also found to be down-regulated in A4497 VERO cells and SF-VERO cells.

**Table 4:** The expression patterns of selected miRNA in other AGMK cells using Taqman quantitative RT-PCR^1^.

miRNA	CV-1	BSC-1	A4497 p165	A4497 p>200	SF-VERO
miR-376a	1.0	3.8	10.8	28.3	52.8
miR-654-3p	1.3	4.9	10.2	32.4	20.2
miR-543	1.4	10.6	8.9	34.1	27.9
miR-382	1.0	2.2	4.1	ND	12.2
miR-31	−65.0	−65.0	−290.0	−1942.0	−345.0
miR-200c	−1.5	−1.3	−8.8	−10.0	−10.3
miR-218	−3.4	−1.0	−10.0	−19.8	−6.7
miR-183	−5.9	−5.5	−5.3	−2.2	−33.0
miR-103	1.0	1.2	1.0	-2.2	1.0
miR-25	1.0	1.0	1.0	1.6	1.1

1 Results from the triplicate samples were averaged following normalization to the levels of small RNA U6B. The data were presented as fold change over pAGMK miRNA levels.

ND: not determined.

We next evaluated the non-tumorigenic CV-1 and BSC-1 cell lines (Table 1) for their cell-growth rates, their wound-healing and invasion characteristics, and their patterns of expression of the same selected miRNAs. These cell lines were independently derived from pAGMK cells [32], [33] and have no lineage relationship to VERO cells. Similar to 10-87 LP cells, CV-1 and BSC-1 cells exhibited limited motility and invasiveness (Fig. 1B and Fig. 2B, respectively). In contrast to 10-87 LP cells, the expression patterns of the selected miRNAs in CV-1 and BSC-1 cell lines were somewhat variable. The expression levels of the miRNAs in CV-1 cells were similar to those in 10-87 LP cells; however, the upregulated miRNA expression levels in BSC-1 cells were slightly higher than those exhibited by 10-87 LP cell and slightly lower than those exhibited by A4497 cells at p165, placing them between 10-87 LP and 10-87 HP (Table 4).The increasing trend in selected miRNA expression by the different AGMK lineages positioned between CV-1 and SF-VERO cells appeared to correlate with the progressive stages of neoplastic development that result in the expression of the tumorigenic phenotype.

### miRNA over-expression promotes cell migration and invasion

**Figure 4:**
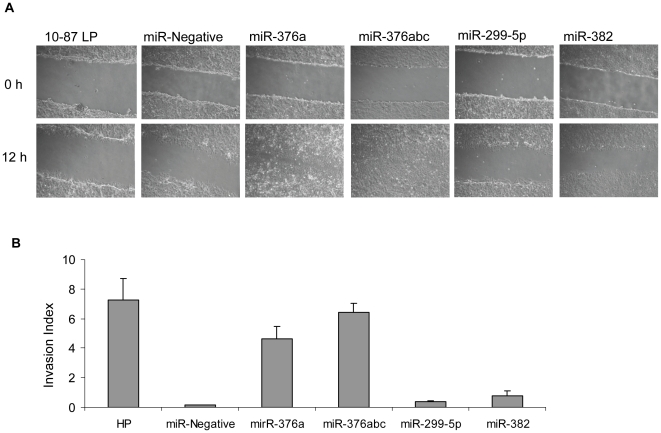
Correlations of over-expression of miRNA with migration and invasive phenotypes. (A) Wound-healing assay of stably expressing miR-376a, miR376abc (a polycistronic cluster of miR-376a, miR-376b and miR-376c), miR-299-5p or miR-negative and 10-87 LP cells; (B) Matrigel-invasion assay with stably expressing miR-376a, miR-376abc, miR-299-5p or miR-negative 10-87 HP cells. 10-87 LP cells were used as the control in the computation of the invasion index.

To determine whether the overexpression of the identified signature miRNAs could confer phenotypic changes, stable cell lines were created by cloning the pre-miRNA sequences of miR-376a, miR-376abc, miR-299, or miR-382 into the pcDNA6.2-GW/EmGFP-miR vector, which contains a green fluorescent protein (GFP) cassette, and introducing them into the non-tumorigenic 10-87 LP cells. We confirmed the expression of each of the miRNAs in their stable cell lines by qRT-PCR. The consequence of miRNA over-expression on the phenotype of the 10-87 LP cells was assessed in the wound-healing and the Matrigel-invasion assays. In the former assay, photomicrographs were taken of wounded cell-culture monolayers at time 0 and at every 3 h up to 15 h to determine the effect of overexpression of the miRNAs on the rate of migration. Only photomicrographs at 0 and 12 h are shown (Fig. 4A). The cells expressing miR-376a or miR-376abc displayed an increase in migration compared with control 10-87 LP cells. However, little or no migration was observed for cells expressing miR-299 or miR-382. In agreement with the wound-healing assay, over-expression of miR-376a or miR-376abc also resulted in more than a four-fold increase in invasiveness, whereas miR-299 or miR-382 had no effect (Fig. 4B). The miRNA effect was specific, as we found no change in migration or invasion ability of cells expressing irrelevant miRNA (miR-control) in 10-87 LP cells. These results demonstrated that over-expression of some of the identified signature miRNAs were able to confer the migration and invasion phenotypes of 10-87 HP cells to 10-87 LP cells.

### Chromosomal location of differentially expressed miRNAs

Using the Sanger miRNA database, we examined the chromosomal distribution of miRNAs that were altered in 10-87 LP and 10-87 HP cells. miRNA genes are frequently located in cancer-associated regions of the human genome [34], and alterations of chromosomal regions containing miRNAs have been reported in human cancers [35], [36]. Because the analysis of the genome of the African green monkey is not complete, we used the rhesus monkey as a guide to examine the chromosomal distribution of the differentially expressed miRNAs. A pattern emerged when the chromosomal location of the genes for those miRNA whose expression was altered in the 10-87 HP cells was determined. The 10 miRNA genes whose expression was up-regulated (Table 2) were clustered within a 47.6 kb region on the rhesus macaque chromosome 7. This chromosomal region is homologous to human chromosomal region 14q32.31, which can be found altered in some human tumors [37], [38]. The panel of the 776 miRNAs screened in this study included other miRNAs whose genes are also located in this region, such as miR-300 and miR-541, but their expression was not modulated with passage of VERO cells. The 10-87 HP VERO cells were also characterized by the down-regulation of 17 miRNAs. For these miRNAs, no clustering of their genes with a specific chromosomal location was observed (Table 2).

## Discussion

Dysregulation of miRNA expression has been linked to the formation and progression of many types of cancers [13], [16], [37], [39], [40], [41], [42], [43]. A number of studies have shown that profiles of miRNA expression can serve as phenotypic signatures of different cancer types. For example, analysis of a panel of more than 200 miRNAs from 334 chronic lymphocytic leukemia samples indicated that miRNA profiles reflected the developmental lineage and differentiation status of this neoplasm [20]. A similar study that involved analysis of 540 solid tumors identified miRNA signatures composed largely of over-expressed miRNAs [44]. In addition, miRNA expression profiling of 60 cancer-derived cell lines (NCI-60 cell lines) allowed the clustering of the cell lines according to their tissue of origin and identified specific miRNAs in different tumor types as candidate oncogenes and tumor-suppressor genes [45]. Another recent study identified miRNA expression patterns that appeared to be associated with steps during *in vivo* neoplastic development in a mouse pancreatic tumor model [46]; this study found that miRNA profiling can be employed to detect miRNA dysregulation at different stages of neoplastic progression *in vivo* (hyper-proliferation, angiogenesis, primary tumor, and metastasis).

**Figure 5:**
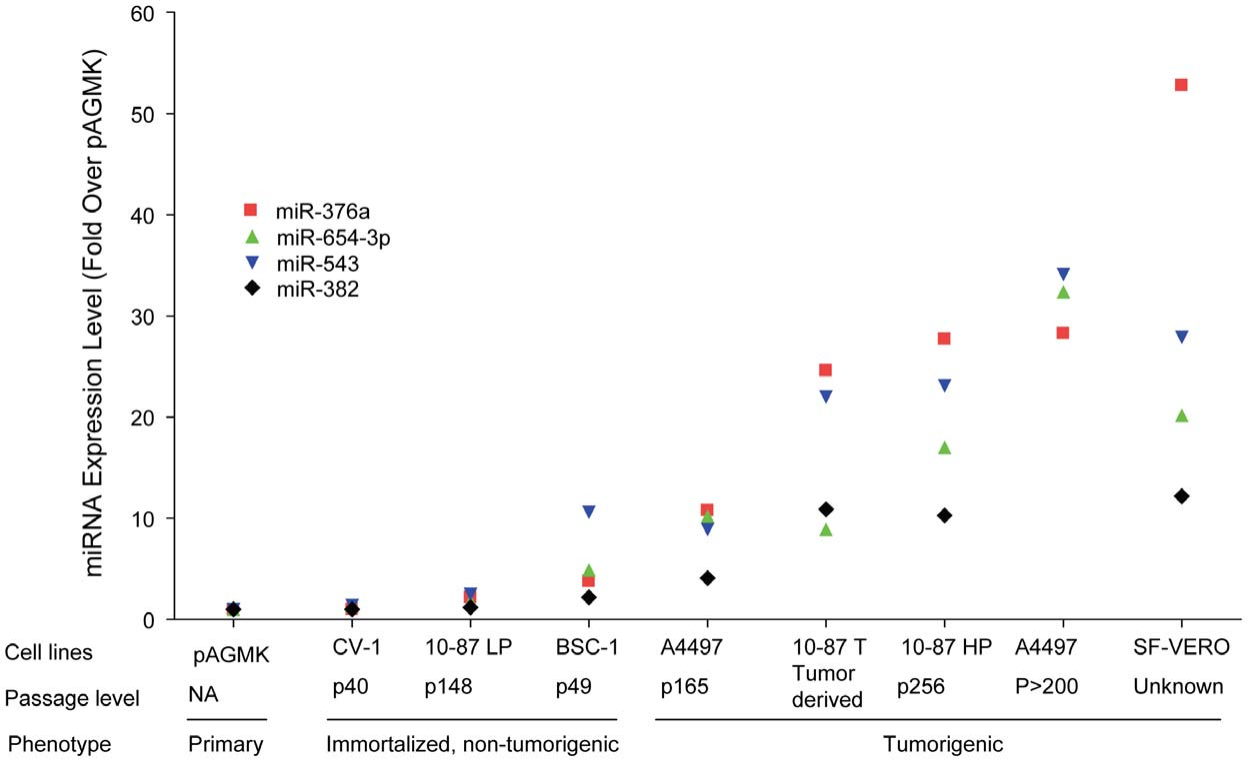
Correlation between expression of miRNAs and the neoplastic phenotype. The qRT-PCR values of over-expressed miRNAs (Table 3) were used to locate on the X-axis the position of seven lines of immortalized AGMK cells relative to pAGMK cells. Based on the relative magnitude of the expression levels of these miRNAs compared with their expression levels in pAGMK cells, the non-tumorigenic CV-1 cells were the closest and the tumorigenic SF-VERO cells were the most distantly removed from pAGMK cells (the reference point for the non-tumorigenic phenotype). When the *in vitro* and *in vivo* characteristics of all cells were superimposed, the trend in miRNA expression in the different AGMK lineages between CV-1 and SF-VERO cells on the X-axis appears to correlate with both the passage level in tissue culture and the evolution of the neoplastic process. The phenotypes were deduced from Table 1 and the data from the invasion assays.

Reported here are the results of the first miRNA expression-profiling study of non-human primate cells that transformed spontaneously during serial passage in cell culture. We evaluated patterns of miRNA expression in pAGMK cells and in derivatives of the 10-87 VERO cell line (10-87 LP cells, 10-87 HP cells, and 10-87 T cells) in an attempt to identify the miRNAs whose altered expression might correlate with, and perhaps be involved in, the evolution of the neoplastic phenotypes that occurred during passage of these AGMK cells in tissue culture. Hierarchical clustering of miRNA expression profiles revealed that many miRNAs were differentially expressed in the tumorigenic 10-87 HP cells and tumor-derived 10-87 T cells when compared with pAGMK cells. In contrast, fewer miRNAs were differentially expressed when the non-tumorigenic 10-87 LP cells were compared with pAGMK cells. These results suggested that the dysregulation of the majority of miRNAs occurred in the 10-87 cell lineage during the transition from a non-tumorigenic to a tumorigenic phenotype. Furthermore, when the expression levels of the up-regulated miRNAs were organized according to their cell line-determined expression patterns, a correlation emerged between the relative expression (compared with levels in pAGMK cells) of representative miRNAs and the evolution of the 10-87 VERO cells into cells that could form tumors in nude mice (Fig. 5). From this correlation, we suggest that a sequence of changes in miRNA expression occurred during the tissue-culture passage-induced neoplastic transformation of 10-87 VERO cells. The initial changes in miRNA expression, which appear to be associated with the initiation of neoplastic development and cell immortalization, occurred prior to p148 (a passage level at which 10-87 LP cells were shown to be non-tumorigenic). These initial changes consisted of the down-regulation of multiple miRNAs, highlighted by the down-regulation of miR-31, miR-200c, and miR-218. The up-regulation of specific miRNAs occurred between p148 and p256 (Table 3); p256 was a passage level at which 10-87 HP cells expressed the ability to form tumors in newborn nude mice. The miRNA-expression patterns obtained from immortalized non-tumorigenic CV-1 cells and BSC-1 cells and the tumorigenic A4497 VERO cells at p165 supported the evolving increase in miRNA expression found between 10-87 LP cells at p148 and 10-87 HP cells at p256 (Fig. 5). In contrast, the pattern of expression of the down-regulated miRNAs did not appear to correlate with the change in VERO cells to a tumorigenic phenotype. In view of the evidence that many tumors occurring in nature arise from the selection of cells with genetic and/or epigenetic changes [3], one interpretation of our data is that the *in vitro* passage of VERO cells selected for the expansion of cells bearing alterations in the expression of specific miRNAs. These findings lead us to propose that the miRNA signatures identified in early-passage VERO cells (10-87 LP) and late-passage VERO cells (10-87 HP and 10-87 T cells) were indicative of the progressive changes in VERO cells that were undergoing neoplastic transformation during serial passage in cell culture.

To determine whether the overexpression of the identified signature miRNAs could increase the levels of cell migration or invasion – phenotypes that correlated with the capacity to form tumors in animals – stable cell lines expressing the pre-miRNA sequences of some miRNAs were generated from the non-tumorigenic 10-87 LP cells. These experiments demonstrated that miR-376a and miR-376abc were sufficient to confer the cell migration and invasion phenotype. No change in migration and invasion phenotypes was observed in cells expressing miR-382 or miR-299-5p. Recently, a target and function of miR-376a has been identified. A human cytomegalovirus (HCMV) miRNA acts synergistically with a cellular miR-376a to suppress one of the major histocompatibility complex class I polypeptide-related sequences (MICB) during HCMV infection [47]. The MICB is a known ligand for natural killer cell activating receptor NKG2D, which is over-expressed on cell surfaces after stress such as that induced by viral infection and cell transformation [48], [49]. It has been documented that overexpression of microRNAs binding to the MICB 3′ UTRs results in enhanced tumorigenicity through the downregulation of MICB expression, which enables tumors to avoid immune recognition [47], [48], [49], [50]. Furthermore, it was found that NKG2D-mediated tumor rejection can be effective at early stages of tumor growth [49], [51], [52], [53]. While the specific effect of miR-376a on tumorigenicity has not yet been demonstrated, it is plausible to suggest that the 10-87 VERO cells might use a similar mechanism as does HCMV to escape the immune system during tumor growth in athymic nude mice.

When we compared experimental results from several published studies on the differential expression of miRNA in different cancers with our results, 30% (18 miRNAs) of the dysregulated miRNAs identified in our study (Fig. 3) have an association with a variety of other cancer types, including renal, prostate, lung, bladder, breast, ovarian and retinoblastoma [16], [34], [41], [42], [44], [45], [46], [54], [55], [56], [57], [58], [59], [60], [61], [62], [63], [64], [65], [66], [67], [68], [69]. When compared with expression profiles of miRNAs associated with renal cancer, there was a 15% overlap with the miRNAs described in a recent report [54]; however, only miR-376a [67] out of the highly upregulated miRNAs, and miR-638, miR-200b and miR-200c from the highly downregulated miRNAs, were found to be in common between our studies and other studies [67], [70], [71], [72]. Although, there are differences in our *in vitro* model of neoplastic development in VERO cells, which were derived from the kidney of African green monkey, and tumors of the kidney that originated *in vivo* in humans, the finding of similar patterns of dysregulation of miRNAs in both systems suggests that similar molecular processes may be underway during neoplastic development in cells from both species irrespective of their microenvironments.

The other observation was the chromosomal clustering of the genes for some of the miRNAs up-regulated in 10-87 HP cells. Importantly, all of the genes for the 10 up-regulated miRNAs in 10-87 HP cells are clustered in a homologous chromosome region of rhesus monkeys and humans. The genes for the 10 miRNAs highly expressed in 10-87 HP cells and 10-87 T cells are spread over 47.6 kb on rhesus chromosome 7. This highly conserved chromosomal region is homologous to human 14q32.31. Interestingly, specific up-regulation of some miRNAs whose genes are located in the human 14q32 imprinted domain has been reported in acute myeloid leukemia [37]. Clustering of these miRNA genes suggests that their expression may be co-regulated and that they might play a role in a common molecular process. However, genes for some miRNAs included in the same region did not show an increased expression in 10-87 HP cells, suggesting a complex mechanism of gene regulation in this region. It has been suggested that some miRNA genes in this region act as tumor-suppressor genes and that changes in the methylation status of their promoters can trigger cancer development [36], [37]. Furthermore, epigenetically regulated expression of miRNA genes associated with oncogenic activity has been described for some miRNAs [18], [73], [74]. Thus, it is possible that the increased expression of some miRNAs but not others in the cluster in 10-87 HP VERO cells could be due to epigenetic changes within this chromosomal region. Another possibility is that the altered level of mature miRNA could be due to differential miRNA processing in 10-87 LP cells and 10-87 HP cells, as reported in different stages of colorectal neoplasia [75]. Studies are being initiated to evaluate the mechanism of the regulation of these miRNAs.

The phenomena of neoplastic development and neoplastic transformation, whether they occur *in vivo* or *in vitro*, are thought to represent the accumulation of a series of genetic and epigenetic alterations that disrupt the normal processes of cell division and tissue integrity [1], [2], [3], [4]. These processes convey upon the cells in which they occur the propensity to outgrow unaffected cells and either expand locally to produce tumor masses or spread and gradually replace unaffected cells in tissue cultures. As these genetic and epigenetic alterations accumulate, the affected cells assume further growth advantages that allow them to invade local tissues in their host or overgrow unaffected cells *in vitro*, at some point producing cells that form invasive tumors or cells that possess the capacity to form tumors when injected into animals. In the VERO cell model, we have found that miRNA down-regulation occurs during the initial stages of neoplastic transformation that are associated with immortalization and some yet-to-be defined forms of neoplastic progression that are not reflected in a currently detectable phenotype by VERO cells at p140. miRNA up-regulation occurs as the immortalized cells are undergoing further neoplastic progression through the conversion, during 116 passages (from p140 to p256) in cell culture, to the expression of a tumorigenic phenotype. We have identified miR-376a and the polycistronic miR-376abc as having a functional role in establishing the enhanced cell migration and cell invasion phenotypes, which are correlated with the expression of the VERO cell tumorigenic phenotype. Studies are underway to determine whether one or more of these over-expressed miRNAs can confer on non-tumorigenic VERO cells the capacity to form tumors in animals and consequently can be used as biomarkers for the expression of the VERO-cell tumorigenic phenotype.

The associations between neoplastic processes and miRNA dysregulation in the VERO-cell model presented in this report provide additional support for the concept that neoplastic development *in vitro* and *in vivo* have similarities. If further evidence for these similarities is forthcoming, the VERO-cell model may provide a format for the systematic comparison of neoplastic development *in vivo* and neoplastic transformation *in vitro* across tissues, organs, and species.

## Materials and Methods

### Cell lines and cell culture

Cells from the World Health Organization (WHO) VERO cell bank 10–87 (ATCC 10–87 VERO cells) were serially passaged in Dulbecco's Modified Eagle's Medium (DMEM) containing 10% fetal bovine serum (FBS) (DMEM-10) from p134 to p250 by sub-culturing VERO cells before they reached confluence [11]. For the studies in this report, ATCC 10-87 VERO cells at p148 and p256 were designated as low passage (10-87 LP) and high passage (10-87 HP) VERO cells, respectively. A4497 VERO cells were obtained from J.G. Tully, NIAID, and a cell bank was established at p160. Serum-free (SF) VERO cells were provided by J. Weir (DVP, OVRR, CBER); the passage level of this cell line is unknown. BSC-1 and CV-1 cells were obtained from ATCC at passage 43 and 34, respectively. The control cells were from 3 different lots (obtained from the kidneys from 3 different monkeys) of pAMGK cells and were obtained from Diagnostic Hybrids (Athens, Ohio). A cell line (10-87 T) was established from a tumor formed in a newborn nude mouse following subcutaneous inoculation of 10-87 HP cells [11]. The cells used in this study were confirmed to be of simian origin by karyotyping (10-87 LP, 10-87 HP, and 10-87 T) and by PCR using primers that recognize simian SINE sequences. The ability of these cells to form tumors *in vivo* is summarized in Table 1.

### Determination of cell-growth curves

Dishes (60 mm) were seeded with 5×10^4^ cells in DMEM-10. The cells were trypsinized and counted in triplicate (using trypan-blue exclusion to determine the number of viable cells) after 16, 24, 48, and 72 h in culture using a Cellometer (Nexcelom Biosciences, Lawrence, MA). Growth curves of the VERO cells were plotted from successive cell counts as described [76].

### RNA extraction and qRT-PCR assays

Total RNA from pAGMK cells and the cell lines was extracted and purified using the miRNeasy mini kit according to the manufacturer's procedures (Qiagen Inc., Valencia, CA). Taqman miRNA assays (Applied Biosystems, Foster City, CA) were used to quantify mature miRNA expression as described [77]. For miRNA expression quantification, each reverse transcriptase (RT) reaction volume of 20 µL consisted of 50 ng of purified total RNA, 1× RT buffer, dNTPs (each at 0.375 mM), 5 U/µL MultiScribe reverse transcriptase, 50 nM stem-loop RT primers and 0.38 U/µL RNase inhibitor (Applied Biosystems). RT reactions were incubated at 16°C for 30 min, 42°C for 30 min and 85°C for 5 min. Real-time PCR amplifications were performed in triplicate in 10-µL volumes. Quantitative miRNA expression data were acquired and analyzed using an ABI Prism 7900 Sequence Detection System (Applied Biosystems). Experiments were done in triplicate and an average was calculated. Expression values were normalized to a small nucleolar RNA, RNU6 (Applied Biosystems). ΔCt values were calculated using the Ct values of the miRNA probes and the RNU6 for each corresponding sample. ΔΔCt values are calculated using the ΔCt values of the pAGMK cells and the experimental cell lines for each miRNA probe. The fold change over pAGMK was calculated.

### MicroRNA microarray assay

The microarray-expression assay was performed using a service provider (LC Sciences, Houston, Texas). Because the genome sequence of the African green monkey is not available, there are no commercially available African green monkey miRNA chips. A primate miRNA chip that contained all human and non-human primate miRNA transcripts in the Sanger miRBase Release 10.0 was used (http://microrna.sanger.ac.uk/). Total RNA (4 to 8 µg) was size fractionated using a YM-100 Microcon centrifugal filter (Millipore, Billerica, MA), and the small RNAs (<300 nucleotides) were isolated and 3′ extended with a poly(A) tail using poly(A) polymerase. An oligonucleotide tag was then ligated to the poly (A) tail for later fluorescent-dye staining. Hybridization was performed overnight on a µParaflo microfluidic chip using a micro-circulation pump (Atactic Technologies, Houston, TX). The hybridization buffer was 6xSSPE (0.90 M NaCl, 60 mM Na_2_HPO_4_, 6 mM EDTA, pH 6.8) containing 25% formamide at 34°C. On the microfluidic chip, each detection probe consisted of a chemically modified nucleotide-coding segment complementary to target miRNA (http://microrna.sanger.ac.uk/sequences/) and a spacer segment of polyethylene glycol to extend the coding segment away from the substrate. The detection probes were made by *in situ* synthesis using photogenerated reagent (PGR) chemistry. The hybridization-melting temperatures were balanced by chemical modifications of the detection probes. After RNA hybridization, tag-conjugating Cy3 and Cy5 dyes were circulated through the microfluidic chip for dye staining. Fluorescence images were collected using a laser scanner (GenePix 4000B, Silicon Valley, CA) and digitized using Array-Pro image analysis software (Media Cybernetics, Bethesda, MD). Data were analyzed by first subtracting the background and then normalizing the signals using a LOWESS filter (Locally-weighted Regression) [78] to balance the intensities of the Cy3- and Cy5-labeled transcripts so that differential expression ratios can be calculated. The ratio of the two sets of detected signals for each of the10-87 cells *vs*. the pAGMK cells (log_2_ transformed, balanced) and *p*-values of the *t-*test were calculated; differentially detected signals were those with *p*<0.01. The expression patterns of unfiltered data (the raw data from 654 miRNA data sets) were assessed using unsupervised hierarchical clustering of samples based on average linkage and Euclidian distance. When comparing two groups, the findings were considered significant if: 1) the fold change was ≥4, 2) the *t*-test, *p*-value was <0.01, and 3) the difference in fluorescence intensity between the two group means were >500 arbitrary units. ANOVA was done between the four groups of cell lines. All significantly expressed miRNAs with mean intensity value more than 500 (arbitrary units) in at least in one of the four groups and a *p*<0.01 were selected and transformed to z-intensity values for inclusion in the heat-maps. The clustered heat-map was created in TM4 microarray software (http://www.tm4.org) using the z-transformed miRNA expression. The dendrogram along the rows was calculated using 1-Pearson correlation for the distance measure and complete linkage for the clustering algorithm. Microarray data comply with MIAME requirements and have been deposited at Gene Expression Omnibus data base (accession number GPL10649).

### Wound-healing assay

The wound-healing assay was carried out as described [31] with minor modifications. Cells were seeded in 60-mm dishes (10^5^ cells/dish). When the cultures reached 90% confluence, they were serum starved for 8 h. After scratching the monolayer with a pipette tip, cells were washed with PBS, cultured in DMEM-10, and photographed under a 10× objective every 3 h for the first 15 h and then at 24 h.

### Matrigel-invasion assay

The assay was performed as described [31] with minor modifications. Cells (4×10^4^) in DMEM with 1% FBS (DMEM-1) were placed in the upper chamber of a Biocoat Matrigel-invasion chamber (BD Biosciences, Bedford, MA), and DMEM-10 was used as the chemoattractant in the lower chamber. After incubation for 18 h at 37°C, the filters were stained with Diff-Quick stain set (Dade Berhring Inc., Newark, DE), and the number of cells that penetrated the Matrigel and the control insert were counted with a microscope using a 20× objective (5 randomly selected fields).

### Constructs and stable cell lines

The double-stranded oligonucleotides encoding the pre-miRNA sequences of miR-376a, miR-299, miR-382 or irrelevant miRNA (a miR-negative control, predicted not to target any known vertebrate gene) were cloned into the pcDNA6.2-GW/EmGFP-miR expression vector such that the pre-miRNA insertion site was in the 3′ untranslated (3′UTR) region of the green fluorescent protein (GFP) gene as per the instruction manual (Invitrogen, Carlsbad, CA). The following oligonucleotides were used to generate the pre-miRNAs: miR-376a sense, 5′-tgctGTAAAAGGTAGATTCTCCTTCTATGAGTACATTATTTATGATTAATCATAGAGGAAAATCCACGTTTTC-3′ and miR-376a anti-sense 5′- cctgGAAAACGTGGATTTTCCTCTATGATTAATCATAAATAATGTACTCATAGAAGGAGAATCTACCTTTTAC-3′. miR-299-5p sense, 5′- tgctGAAGAAATGGTTTACCGTCCCACATACATTTTGAATATGTATGTGGGATGGTAAACCGCTTCTT-3′ and miR-299-5p anti-sense 5′-cctgAAGAAGCGGTTTACCATCCCACATACATATTCAAAATGTATGTGGGACGGTAAACCATTTCTTC-3′, miR-382 sense 5′- tgctGTACTTGAAGAGAAGTTGTTCGTGGTGGATTCGCTTTACTTATGACGAATCATTCACGGACAACACTTTTTTCAGTA-3′ and miR-382 anti-sense 5′-cctgTACTGAAAAAAGTGTTGTCCGTGAATGATTCGTCATAAGTAAAGCGAATCCACCACGAACAACTTCTCTTCAAGTAC-3′. An expression plasmid for the polycistronic gene cluster for miR-376a, miR-376b and miR-376c was generated by amplifying 200 bp up-stream and 200 bp down-stream of the genes (termed as miR-376abc) in VERO cell DNA using primers 5′-CCCTCGACGAGAGTGATGGAAGGTGAATC-3′ and 5′-CCAGATCTATACTGAGAACACAGCCTTGT-3′. This PCR product was digested with *Sal* I and *Bgl* II and cloned into the *Sal* I and *Bgl* II sites of the digested pcDNA6.2-GW/EmGFP-miR expression vector in the 3′ UTR region of GFP protein. The plasmids were verified by sequencing across the insert. The expression constructs were introduced into the non-tumorigenic 10-87 LP cells using Lipofectamine 2000 (Invitrogen, Carlsbad, CA). Stable cell lines that constitutively expressed the specific miRNAs were selected using blasticidin (8 µg/mL). Blasticidin-resistant colonies were identified after 15 days of selection. The stable cell lines were sorted for high GFP expression using a BD FACSAria II (BD Biosciences, San Jose, CA) and tracked using fluorescence microscopy.
